# Supplementary figures and images for: Sub-Wavelength Focusing in Inhomogeneous Media with a Metasurface Near Field Plate
Source: Sensors (Basel). 2019 Oct 18;19(20):4534. doi: 10.3390/s19204534 (PMC6832601; doi:10.3390/s19204534)

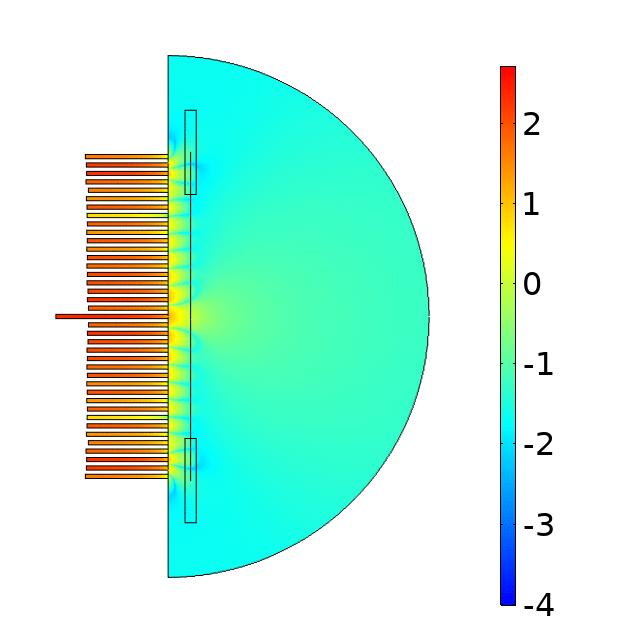

Supplement: Supplementary file 1 [file sensors-19-04534-s001.zip › NFP_Proof_Supp/S1.gif]

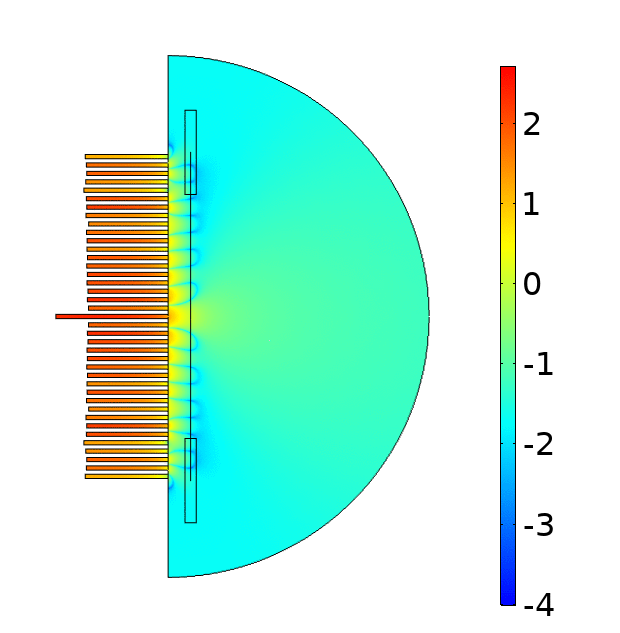

Supplement: Supplementary file 1 [file sensors-19-04534-s001.zip › NFP_Proof_Supp/S2.gif]
